# Fabrication of Antibacterial and Ultraviolet Protective Wool Fabric Using Multi-Walled Carbon Nanotubes Functionalized with Guanidinylated Hyperbranched Polyethyleneimine Derivative

**DOI:** 10.3390/ma18091993

**Published:** 2025-04-28

**Authors:** Nikolaos S. Heliopoulos, Kyriaki-Marina Lyra, Aggeliki Papavasiliou, Fotios K. Katsaros, Kostas Stamatakis, Sergios K. Papageorgiou, Zili Sideratou

**Affiliations:** 1 Institute of Nanoscience and Nanotechnology, National Centre of Scientific Research “Demokritos”, 15310 Aghia Paraskevi, Greece; nikosheliopoulos@gmail.com (N.S.H.); k.lyra@inn.demokritos.gr (K.-M.L.); a.papavasiliou@inn.demokritos.gr (A.P.); f.katsaros@inn.demokritos.gr (F.K.K.); 2 700 Military Factory, Supreme Military Support Command, 18648 Piraeus, Greece; 3 Institute of Biosciences and Applications, National Centre of Scientific Research ‘‘Demokritos”, 15310 Aghia Paraskevi, Greece; kstam@bio.demokritos.gr

**Keywords:** hybrid textiles, wool, carbon nanotubes, hyperbranched polymers, guanidinium groups, bactericidal properties, UV blocking properties

## Abstract

Wool textiles with multifunctional properties such as self-cleaning, antibacterial, electrical conductivity, UV blocking etc. have recently attracted interest. Among the materials employed towards their development, carbon nanotubes (CNTs) have been widely investigated due to their unique chemical, mechanical and electrical properties, exhibiting also notable UV-blocking properties. However, their limited dispersibility in solvents, particularly in water, has hindered their extensive industrial application and diminished their significant potential. In this work, two guanidinylated derivatives of hyperbranched polyethyleneimine (GPEI5k and PEI 25K) functionalized oxCNTs (oxCNTs@GPEI5K and oxCNTs@GPEI5K), with exceptional aqueous compatibility and colloidal stability, developed in our recent publication, were evaluated as to their antibacterial activity on Gram (-) *Escherichia coli* and Gram (+) *Staphylococcus aureus* bacteria and their cytotoxicity against mammalian cells, and the most promising, i.e., oxCNTs@GPEI5K, was subsequently used as finishing agent of wool fabric. The resulting wool textiles were evaluated for color, wash fastness, antibacterial properties, and UV-blocking performance. The GPEI-functionalized oxCNTs derivative, exhibited uniform distribution and good adhesion onto the wool fabrics yielding multifunctional wool fabrics with sustained antibacterial properties even after multiple washing cycles. Additionally, the modified textiles exhibited improved ultraviolet protection, highlighting their potential for multifunctional applications in antibacterial and UV-shielding textiles.

## 1. Introduction

Textiles with multifunctional properties have recently attracted interest in an effort to meet current demands in the field of textiles that include properties such as self-cleaning, antibacterial, electrical conductivity, UV blocking, etc. [1]. The antibacterial properties in particular are of significant importance as microorganisms attracted onto textiles can result in discoloration, unwanted odors, dermal infection and product deterioration, as well as diseases and allergic responses [2]. Thus, the prevention of microbial attack on textiles has become increasingly important to both consumers and producers [3,4].

Carbon nanotubes (CNTs) are among the most resilient materials recognized due to their unique chemical, mechanical and electrical properties. Thus, their functionalization with various polymers or their introduction into polymeric matrices as fillers has been widely investigated yielding hybrid nanomaterials or polymer nanocomposites with superior electrical and mechanical properties, UV and oxidation resistance, etc. [5]. Recently, the broad-spectrum antibacterial activity of functionalized CNTs has also been reported, adding to their potential applications [6,7,8,9,10]. Nonetheless, their limited dispersibility in solvents, particularly in water, has hindered their extensive industrial application and diminished their significant potential. Efforts to address this issue focused on modifying their surface either through various covalent, e.g., by introducing oxygen-containing groups or by grafting with hydrophilic moieties on their surface, or through non-covalent functionalization techniques [11,12,13,14], using various water-soluble polymers [15,16,17,18] or other molecules such as surfactants [19,20].

It is known that wool fabric is a superior textile material due to its durability, warmness, smoothness and other properties like flame, dirt and odor resistance. Additionally, the development of wool textiles with various functional properties such as ultraviolet (UV) resistance, antimicrobial properties and flame retardancy are becoming increasingly popular. Wool coating using CNTs would be a very good candidate towards such multifunctional wool textiles. Several methods of preparing antibacterial wool fabrics have been reported [21,22,23,24], including adsorbing or grafting known antibacterial agents such as silver nanoparticles, metal complex and quaternary ammonium group etc. on the fibers’ surface. However, even though techniques such as spray-coating, dip-coating, drying, or exhaustion-also utilizing various polymers as stabilizers-have been employed for the deposition of carbon nanotubes onto fabrics [25,26,27,28,29], only very few reports can be found on wool finishing using functionalized CNTs [30,31,32,33,34,35,36,37,38], and to the best of our knowledge, none of them refers to CNTs-functionalized wool fabrics with antibacterial and ultraviolet protective properties.

It is well documented that functionalized single-walled carbon nanotubes (SWCNTs) and multi-walled carbon nanotubes (MWCNTs) demonstrate notable antibacterial properties against both Gram (-) and Gram (+) bacteria [9,39,40,41,42]. The characteristics of CNTs, including their structural properties [43,44], dispersion concentration [45], degree of aggregation [46], surface functionalization [7,47,48,49], have been shown to affect their final antibacterial properties. In this regard, the dispersibility in water can significantly affect antibacterial effectiveness, since well-dispersed CNTs improve their interaction with cells, resulting in enhanced antibacterial characteristics. In fact, it was found that well-dispersed CNTs were more harmful to bacteria compared to CNT aggregates due to an increased interaction with bacterial cells [15,20].

In our recent publication [50], oxidized MWCNTs (oxCNTs) were functionalized with guanidinylated derivatives of hyperbranched polyethyleneimine having 5000 and 25,000 Da molecular weight (GPEI5K and GPEI25K) yielding two nanohybrid materials (oxCNTs@GPEI5K and oxCNTs@GPEI25K) with exceptional aqueous compatibility and colloidal stability that can be used as efficient nanoscale doxorubicin delivery system with high selectivity against cancerous cells. On the other hand, in our previous publication [51], we have proved that these GPEI derivatives provide enhanced antibacterial activity to oxidized carbon nanodisks (oxCNDs) due to the polycationic character of the produced oxCNDs@GPEIs nanohybrids that enables effective interaction with the bacteria. Thus, in this study, GPEI-functionalized oxCNTs were initially assessed regarding their antibacterial properties against Gram (-) bacteria (*Escherichia coli*) and Gram (+) bacteria (*Staphylococcus aureus*), while their cytocompatibility was evaluated on mammalian cells. Subsequently, the most promising nanohybrid was applied at various concentrations as novel finishing agent for the preparation of multifunctional wool fabrics. The antibacterial properties of the obtained hybrid textiles (HTs) were studied using a novel in situ quantitative method, which determines the Bacterial Protection Index (*Π*_7_) of materials. The durability of antibacterial finishing was evaluated following three washing treatments (water cleaning, dry cleaning and with liquid CO_2_). Furthermore, the protection ability of HTs against UV radiation was investigated and the performance of the applied finishing for wool fabric was determined.

## 2. Materials and Methods

### 2.1. Chemicals and Reagents

Commercial undyed 100% wool plain weave fabric (Hellenic Fabrics SA, Giannitsa, Greece) having a mass/unit area of 155 g·m^−2^ was used for antibacterial finishing. The density and yarn count of both warp and weft are 23 ends∙cm^−1^ and 32.5 Tex, respectively. The fabrics were free from residual chemicals or finishing agents. Multi-walled carbon nanotubes (CNTs) containing carboxyl groups >8% *w*/*w* (oxCNTs), hyperbranched polyethyleneimine (PEI) of 5000 Da (PEI5K, Lupasol^®^ G100, water-free, 99%) and 25,000 Da molecular weight (PEI25K, Lupasol^®^ WF, water-free, 99%) were donated by Glonatech S.A (Athens, Greece) and BASF (Ludwigshafen, Germany), respectively. The oxCNTs purity is >97% while their mean diameter is 20 ± 5 nm and their length is lower than 5 µm. Levantin LNB (non-ionic washing agent) was purchased from BASF (Athens, Greece). 1*H*-Pyrazole-1-carboxamidine hydrochloride, *N*,*N* diisopropylethylamine (DIPEA), thiazolyl blue tetrazolium bromide (MTT), dialysis tubes (M.W. cut-off: 1200), sodium chloride (NaCl), tryptic soy broth (TSB), resazurin, glutaraldehyde (solution, 25%) and sodium cacodylate were purchased from Sigma-Aldrich Ltd. (Poole, UK). D-MEM low glucose with phenol red, fetal bovine serum (FBS), penicillin/streptomycin, L-glutamine, phosphate buffer saline (PBS) and trypsin/EDTA were purchased from Biochrom (Berlin, Germany). Peptone from Casein was obtained from AppliChem GmbH (Darmstadt, Germany), while agar and lambda broth (LB) were purchased from MP Biomedicals (Illkirch, France). Solvents with high purity such as isopropanol, DMF, perchloroethylene, ethanol and methanol as well as acetic acid (100%) were obtained from Merck KGaA (Calbiochem^®^, Darmstadt, Germany).

### 2.2. Preparation of GPEIs Functionalized oxCNTs

**Scheme 1:**
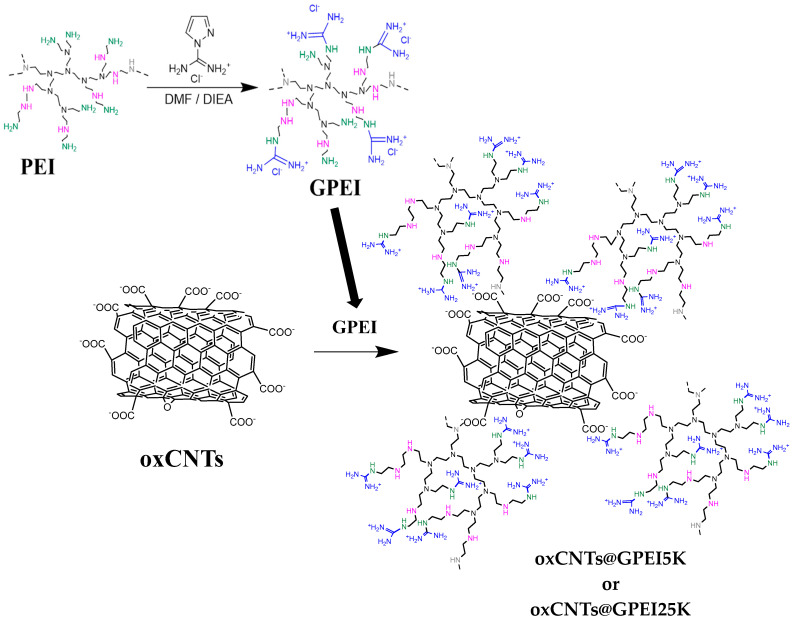
Schematic representation of the synthesis of GPEI (1st step) and subsequently of GPEI-functionalized oxCNTs (2nd step).

GPEIs functionalized oxCNTs were synthesized as described in our previous publications [50]. In brief, in the first step (Scheme 1), hyperbranched polyethyleneimines (PEI) having molecular weights of 5000 or 25,000 Da were functionalized at the primary amino groups with guanidinium groups following a known guanidylation reaction, affording two guanidinylated PEI derivatives, i.e., GPEI5K and GPEI25K [50,52]. Specifically, 0.01 mmol of PEI5K or PEI25K dissolved in DMF (10 mL), and then were added to a 10 mL DMF solution containing 0.4 mmol or 2 mmol 1*H*-Pyrazole-1-carboxamidine hydrochloride and 0.8 mmol or 4 mmol DIPEA, for the two molecular weights respectively. The reaction mixture was stirred at room temperature in inert atmosphere for 24 h. Then the crude products were obtained after precipitation in diethyl ether and subsequent vacuum drying. Finally, the guanidinylated products, GPEI5K and GPEI25K, were received after purification by dialysis against distilled water and lyophilization. The chemical structures of the polymers were established by ^1^H and ^13^C NMR spectroscopy, using a Bruker Avance DRX spectrometer operating at 500, and 125.1 MHz (Bruker Biospin, Rheinstetten, Germany), respectively, at 25 °C, and D_2_O as solvent. The successful introduction of guanidinium groups to PEI was confirmed by a new peak at 3.25 ppm, presented in the ^1^H NMR spectra of both PEI derivatives (Figure S1), assigned to the protons of α methene groups close to guanidinium groups as well as to the new peaks, observed in the ^13^C NMR spectra of both PEI derivatives (Figure S2), at: (i) 157.0 ppm, assigned to the guanidinium group carbon, and (ii) 39.4 and 40.5 ppm attributed to the α-CH_2_ groups close to the guanidinium groups, respectively. Comparing the integration of the peaks at: (i) 3.25 ppm and (ii) 2.70–2.50 ppm, attributed to the protons of methyl groups of PEI scaffold, the degree of functionalization of PEI5K and PEI25K was found to be 98% and 95% substitution degree of the primary amino groups, respectively.

In the second step (Scheme 1), these guanidinylated PEI derivatives strongly interacted with oxidated multi-walled carbon nanotubes though electrostatic forces as well as through hydrogen bonding and van der Waals attraction forces, yielding the nanohybrids oxCNTs@GPEI5K and oxCNTs@GPEI25K. In more details, 50 mg oxCNTs were dispersed in 50 mL distilled water with the aim of a Hielscher UP200S high intensity ultrasonic processor (Hielscher Ultrasonics GmbH, Teltow, Germany), coupled with a sonotrode having a 3 mm diameter tip (50% amplitude, 0.5 cycles/s) for 30 min. Subsequently, the pH of the dispersion was adjusted to ~9 by adding NaOH solution (0.5 M) and then left under stirring for 24 h. 150 mg of GPEI5K or GPEI25K dissolved in 50 mL distilled water was drop-wise added to the as prepared oxCNTs dispersion and stirred for further 48 h, at room temperature. The final nanohybrids materials, oxCNTs@GPEI5K or oxCNTs@GPEI25K, were received after centrifugation at 25,000× *g*, washed until the pH of the supernatant reached the value of 6.5–7.0 and lyophilization. These guanidinylated oxCNTs derivatives were exhaustively physicochemically characterized as described in our recent publication [50], using various techniques, such as FTIR, Raman, XPS, NMR, etc., confirming the successful interaction of GPEIs with oxCNTs nanotubes though electrostatic forces as well as through hydrogen bonding and van der Waals attraction forces. The GPEI content in the final nanohybrids was determined by elemental analysis, using the nitrogen mass fraction measured for each nanohybrid and taken into account that the nitrogen signal mainly originates from GPEI derivatives, as described in our previous publication (See Supporting Information) [50]. Based on the obtained results (Table S1), it was found that the GPEI loading in oxCNTs@GPEI5K and oxCNTs@GPEI25K was 22.7% and 27.5%, respectively.

### 2.3. Antibacterial Evaluation of GPEIs Functionalized oxCNTs

The antibacterial activity of oxCNTs@GPEIs was evaluated on Gram (-) *Escherichia coli* bacteria (*E. coli* DH5α strain ATCC-BAA-3219) and Gram (+) *Staphylococcus aureus (S. aureus* strain ATCC 25923) bacteria, obtained from the American Type Culture Collection (ATCC, Manassas, VA, USA). *E. coli* bacteria were incubated in LB (Luria-Bertani) medium for 18 h, while *S. aureus* bacteria were incubated in tryptic soy broth (TSB) for 16 h. Both strains were incubated in a Stuart SI500 orbital shaker (~200 rpm shaking speed) at 37 °C in aerobic conditions. Then, bacteria suspensions with concentration of ~10^8^ CFU/mL were obtained by adjusting the suspensions optical density (OD) at 600 nm to that of the 0.5 McFarland standard solution [53], employing a Cary 100 Conc UV–visible spectrophotometer (Varian Inc., Mulgrave, Australia) and used for the followed experiments.

The Minimum Inhibitory Concentrations (MIC) of oxCNTs@GPEIs for *E. coli* and *S. aureus* bacteria was determined by the broth micro-dilution method using resazurin as bacterial health indicator according the M07-A9 protocol issued by the Clinical Laboratory Standards Institute (CLSI) [54]. Bacteria suspensions of *E. coli* and *S. aureus* (10 μL) at a final concentration of ~10^5^ CFU/mL were mixed with 990 μL dispersions of oxCNΤs@GPEIs at final concentrations ranging from 10 to 200 μg/mL. The assay was performed in a 96-well plate format in a 100 μL final volume per well. After a 24-h incubation period at 35 °C (*E. coli*) or 37 °C (*S. aureus*) in aerobic conditions, 5 μL of resazurin solution (6.7 mg/mL) were added to each well and bacteria were further incubated for 4 h. Untreated bacteria were used as positive control, while LB or TSB media without bacteria were used as negative control and the fluorescence intensity of the produced resorufin was measured by an Infinite M200 plate reader (Tecan group Ltd., Männedorf, Switzerland, λ_ex_ = 530 nm, λ_em_ = 590 nm). Minimum inhibitory concentrations (MICs) were determined as the lowest concentration at which no fluorescence intensity was recorded, indicating that no bacterial growth occurred.

The colony-counting method was used to assess the minimum bactericidal concentration (MBC) of oxCNDs@GPEIs according the M26-A protocol issued by the Clinical Laboratory Standards Institute (CLSI) [55]. Stock dispersions (5 mg/mL) of each sample were freshly prepared in sterilized water, which were serially diluted in order to obtain dispersions with concentrations ranging from 10 to 400 μg/mL. Then, each dispersion (3 mL) was mixed with the bacteria suspension (10 μL), and incubated on a Stuart SI500 orbital shaker (~200 rpm shaking speed) at 37 °C for 24 h. After a 24 h incubation period, the dispersions were serially diluted with the appropriate medium. Then,100 μL of each dilution was spread onto the surface of LB or TSB agar plates, supplemented with 1.5% *w/v* agar in 90 × 15 cm round plates, and incubated at 37 °C in aerobic conditions. After 18 h of incubation time for *E. coli* or 16 h for *S. aureus*, the colonies on the plates were counted and compared to the control. Untreated bacteria were used as control, following the same procedure. All tests were repeated at least three times. The MBC value was considered to be the concentration at which a 3-log reduction in the viability of the parent bacterial inoculum had occurred.

The morphology of *E. coli* bacteria after 12-h incubation time with oxCNTs@GPEIs was studied by scanning electron microscopy (Jeol JSM 7401F Field Emission SEM, JEOL Ltd., Tokyo, Japan) following an analogous procedure as descripted in our previous publications [56,57]. In brief, bacteria were incubated with oxCNTs@GPEIs at their ½ MIC for 12 h, fixed with a 3% *w/v* solution of glutaraldehyde in sodium cacodylate buffer (100 mM, pH = 7.1), transferred to a glass cover slip coated with poly(L-lysine), dehydrated using an ethanol gradient (twice of 50%, 70%, 95%, and 100% ethanol for 10 min each), dried, and coated with gold in a sputter coater.

### 2.4. In Vitro Cytotoxicity Studies

Cos-7 cells (African green monkey kidney cells) were obtained from the American Type Culture Collection (ATCC, Manassas, VA, USA). The cells were cultured in D-MEM supplemented with 10% FBS, penicillin (100 U/mL)/streptomycin (100 μg/mL) solution and L-Glutamine (2 mM). The cells were incubated in a 5% CO_2_ humidified atmosphere at 37 °C and sub-cultured twice a week after detaching with trypsin (0.05% *w*/*v*)/EDTA (0.02% *w*/*v*) solution. All treatments were performed in complete medium.

The cytotoxicity of oxCNTs@GPEIs was assessed using the well-known MTT assay. Cos-7 cells were inoculated (10^4^ cells/well) in 96-well plates and incubated in complete media (D-MEM) for 24 h. Then, cells were treated with oxCNTs@GPEIs dispersed in D-MEM at various concentrations close to MIC values. After a 24-h incubation time, cell medium was removed and 100 μL of MTT solution (10 μg/mL in D-MEM) was added to each well. After a 4-h incubation time, the produced formazan crystals were solubilized in isopropanol (100 μL/well) and the absorbance was measured at 540 nm using an Infinite M200 microplate reader (Tecan group Ltd., Männedorf, Switzerland). The relative cell viability was determined as percentage compared to untreated cells (control). Blank values measured in wells with isopropanol or no cells were in all cases subtracted.

### 2.5. Wool Fabric Finishing Using oxCNTs@GPEI5K Nanohybrid

Wool fabric was washed in a bath containing 1.0% non-ionic washing agent Levantin LNB at a 30:1 liquor-to-fabric ratio for 15 min at 40 °C. The pH was adjusted at 4.5 by addition of acetic acid solution (10 g/L). The fabric was subsequently rinsed with warm bi-distilled water (40 °C) for 3 min and then with bi-distilled water (25 °C) for 9 min. The samples were then dried at room temperature.

Dry wool fabrics of known dry mass, were placed in 200 mL Erlenmeyer flasks containing aqueous solutions of increasing concentrations of oxCNTs@GPEI5K, at a 100:1 liquor-to-fabric ratio. The flasks were kept for 24 h in an orbital shaker (Julabo SW22, JULABO, Seelbach, Germany) under agitation at 120 rpm at 25 °C. The resulting Hybrid Textiles (HTs) were then rinsed with bi-distilled water (25 °C) for 5 min and dried at room temperature. For comparison reasons, GPEI5K and oxCNTs were also applied following the same procedure.

### 2.6. Determination of Color Strength and Related Parameters

Reflection spectra were recorded for the HΤs and raw wool in the visible range (400–700 nm) by a UV–vis spectrophotometer (Data color SF600 Plus-CT, Datacolor, Basel, Switzerland), using Illuminant CIE *D*_65_ and a 10° observer. Analyses were performed in three different points for each HT, with the average value used for data interpretation.

The color yield *K*/*S* of the fabrics were determined using the Kubelka–Munk equation given below [58]:

(1) $$\frac{K}{S}=\frac{{(1-R_{\lambda_{max}})}^{2}}{2R_{\lambda_{max}}}$$

where *K* is the absorption coefficient, *S* is the scattering coefficient and *R_λmax_* is the decimal fraction of the reflectance value of the HT at peak wavelength.

The relative color strength (*RCS*) between HT_GPEI5K_, HT_oxCNTs@GPEI5K_ and raw wool was obtained using the following relationship [59]:

(2) $$\mathrm{Relative}\;\mathrm{colour}\;\mathrm{strength}\;(\%)=\frac{K/S\;\mathrm{of}\;\mathrm{treated}\;\mathrm{smaple}}{K/S\;\mathrm{of}\;\mathrm{untreated}\;\mathrm{smaple}}\times 100$$

Moreover, the Whiteness Index (*W*) for HT_GPEI5K_ and the color difference (ΔE*) between the HT_oxCNTs@GPEI5K_ samples and raw wool were obtained as follows:

(3) $$W_{10}=Y_{10}+800\left(0.3138-x_{10}\right)+1700(0.3310-y_{10})$$

where *W_10_* is the Whiteness Index, *Y_10_* is the tristimulus value of the specimen and x_10_, y_10_ are the chromaticity coordinates of the specimen [60].

(4) $$\Delta E*=\sqrt{{(\Delta L*)}^{2}+(\Delta a*{)}^{2}+(\Delta b*{)}^{2}}$$

where ΔL* = L*_HT_ − L*_raw wool_, Δα* = α*_HT_ − α*_raw wool_, Δb* = b*_HT_ − b*_raw wool_ and L* describes the lightness, a* measures the redness or the greenness and b* measures the yellowness or the blueness [61].

### 2.7. Wash Fastness Analysis

Three washing procedures (two based on standard methods and one developed *in-house*) were used to evaluate the durability of the HTs antimicrobial finishing upon repeated washings. Standard soap and perchloroethylene solvent were used as indicated by the ISO 105-C10 (Test No B2) [62] and ISO 105-D01 [63] standard methods, respectively. The samples were washed in a Rotawash M228-SDL International machine. The washings were repeated 5 times for both methods.

For the third procedure, fabric samples were washed with liquid carbon dioxide (liqCO_2_). Specifically, samples were soaked in liquid CO_2_ at room temperature and were kept under constant CO_2_ flow of 0.8 mL/min for 25 h. Total washing time was equivalent to fifty cycles of 30-min each.

As carbon dioxide is non-toxic and non-flammable it provides a good alternative to potentially toxic and environmentally harmful solvents such as perchloroethylene or other hydrocarbon solvents used in dry cleaning procedures. Moreover, CO_2_ can be recovered, recycled and reused and as carbon dioxide evaporates during depressurization of the cleaning-vessel, an additional drying stage is not necessary.

The estimation of the color change of the samples was performed according to ISO 105-A02 [64].

### 2.8. Measurement of Antibacterial Properties of HTs

Antibacterial properties of the as prepared HTs were assessed with a novel method which correlates the increase of Chl *α* fluorescence to *Synechococcus* sp. PCC7942 cell proliferation described elsewhere [65,66]. In brief, the unicellular cyanobacterium *Synechococcus* sp. PCC7942, obtained from the Collection Nationale de Cultures de Microorganismes (CNCM), Institut Pasteur, Paris, France, was used in all antibacterial experiments. Cyanobacterial cells were cultured in BG11 medium [67], illuminated with white fluorescent light providing a photosynthetic active radiation (PAR) of 100 μmol photons∙m^−2^∙s^−1^, and aerated with air containing 5% (*v*/*v*) CO₂ in an orbital incubator (Gallenkamp INR-400) at 31 °C.

The concentration of chlorophyll α (Chl α) was determined in DMF extracts. To prepare the samples, an appropriate amount of *Synechococcus* cells was collected by centrifugation (5000 rpm, 5 min) and resuspended in conditioned BG11, ensuring a Chl α concentration of 52.0 μg/mL. A 0.05 mL drop of the cyanobacterial suspension was placed on each fabric sample, forming a spot with a diameter of less than 3.0 mm. After dark-adapting the samples for 15 min using a suitable clip, Chl α fluorescence was measured with a PEA fluorometer (Hansatech Instruments LTD, Norfolk, UK). During measurements, samples were kept in an incubator under white fluorescent light (100 μE∙m^−2^∙s^−1^) at 31 °C.

The fluorescence value (*F*_0_) of the cyanobacterial colony staining each sample was recorded every 24 h until the cell growth phase was complete. The antibacterial properties of the modified samples were determined by calculating the normalized fluorescence changes using Equation (5) and the *M_ι_* index:

(5) $$M_{\iota }=\frac{F_{0_{i}}-F_{0_{0}}}{F_{0_{0}}}\times 100$$

where, $F_{0_{0}}$ and $F_{0_{i}}$ is the cyanobacteria Chl *α* fluorescence value at zero contact time and after 1, 2, …, i days, respectively.

The material’s antibacterial action is represented by the Bacterial Protection Index (BPI) *Π*_7_, given by the Equation (6):

(6) $$\Pi_{7}=\frac{M_{U_{7}}-M_{T_{7}}}{M_{U_{7}}}\times 100$$

where $M_{U_{7}}$ and $M_{T_{7}}$ is the change in cyanobacterial Chl α *F*_0_ value on the untreated and treated sample respectively after 7 days of incubation [68,69].

Additionally, the morphology of the cyanobacteria grown on HT/oxCNTs@GPEI5K was studied by scanning electron microscopy (Jeol JSM 7401F Field Emission SEM, JEOL Ltd., Tokyo, Japan).

### 2.9. Measurement of UV-Blocking Properties

To determine the HTs UV protection performance, the UPF values of treated and untreated wool fabrics were calculated according to the AATCC Test Method 183 [70]. The fabric samples UPFs were calculated according to Equation (7)

(7) $$UPF=\frac{\sum_{280\;\mathrm{nm}}^{400\;\mathrm{nm}}E_{\lambda }\times S_{\lambda }\times \Delta \lambda }{\sum_{280\;\mathrm{nm}}^{400\;\mathrm{nm}}E_{\lambda }\times S_{\lambda }\times T_{\lambda }\times \Delta \lambda }$$

where *E*_λ_ represents the relative erythemal spectral effectiveness, *S_λ_* the solar spectral irradiance (W·cm^−2^·nm^−1^), *T*_λ_ the average spectral transmittance of nine transmission measurements (measurements were taken at 0°, 45° and 90° for triplicates of each HT sample) and ∆λ the measured wavelength interval (nm).

The percent blocking for UV-A and UV-B is calculated using Equation (8) and Equation (9), respectively:

(8) percent blocking (UV−A)=100%−T(UV−A)

(9) percent blocking (UV−Β)=100%−T(UV−B)

where T(UV-A) and T(UV-B) is the average A-range and B-range ultraviolet transmittance from Equation (10) and Equation (11), respectively, expressed as a percentage:

(10) $$T{\left(\mathrm{UV}-A\right)}_{AV}=\frac{\sum_{315\;\mathrm{nm}}^{400\;\mathrm{nm}}T_{\lambda }\times \Delta \lambda }{\sum_{315\;\mathrm{nm}}^{400\;\mathrm{nm}}\Delta \lambda }$$

(11) $$T{\left(\mathrm{UV}-B\right)}_{AV}=\frac{\sum_{280\;\mathrm{nm}}^{315\;\mathrm{nm}}T_{\lambda }\times \Delta \lambda }{\sum_{280\;\mathrm{nm}}^{315\;\mathrm{nm}}\Delta \lambda }$$

Transmission values of treated and untreated fabrics were obtained using a UV-vis Spectrophotometer Perkin Elmer Lambda 40 (PerkinElmer, Shelton, CT, USA), with Labsphere integrating spheres, and 2 nm wavelength intervals.

## 3. Results and Discussion

### 3.1. Antibacterial and Cytotoxic Evaluation of GPEIs-Functionalized oxCNTs

Oxidized MWCNTs (oxCNTs) functionalized with guanidinylated derivatives of hyperbranched polyethyleneimine of 5000 and 25,000 Da molecular weights (GPEI5K and GPEI25K) were prepared and exhaustively physicochemically characterized as described in our recent publication [50]. The obtained nanohybrid materials, namely oxCNTs@GPEI5K and oxCNTs@GPEI25K, exhibited exceptional aqueous compatibility and colloidal stability due to the presence of guanidinium groups all over the oxCNTs sidewalls and were found to be potential doxorubicin nanocarriers with enhanced cell-penetration capability and high selectivity against cancerous cells. On the other hand, in our previous publication [51], these GPEI derivatives were found to exhibit improved antibacterial properties due to the enhanced electrostatic interaction between the guanidinium moieties and anionic groups located on the bacterial membranes. Furthermore, these GPEI derivatives were shown to provide enhanced antibacterial activity to oxidized carbon nanodisks (oxCNDs) due to the polycationic character of the produced oxCNDs@GPEIs nanohybrids that enables effective interaction with the bacteria. In light of these results, in this study, oxCNTs@GPEI5K and oxCNTs@GPEI25K were initially assessed as antibacterial agents and subsequently, the most promising derivative was applied on wool fabrics as finishing material.

**Figure 1:**
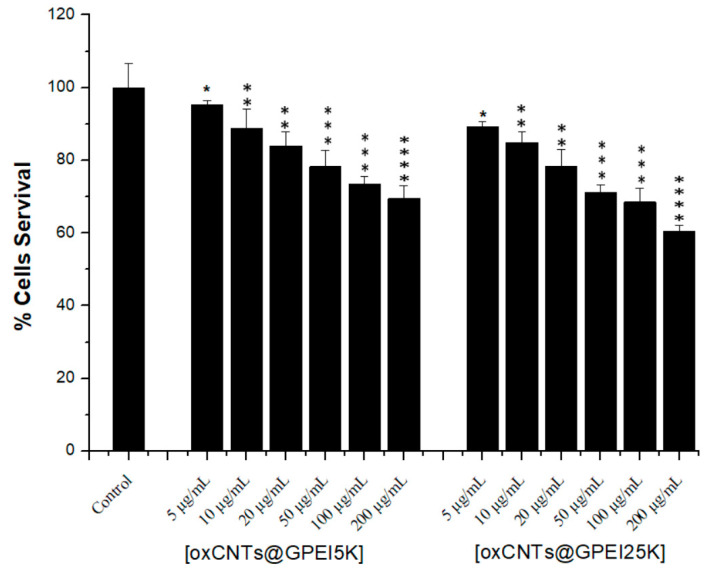
Comparative toxicities of oxCNTs@GPEIs on COS-7 cells after a 24-h incubation time at various concentrations as determined by MTT assays. Data are expressed as mean ± SD of six independent values obtained from at least three independent experiments. The statistical significance, obtained from Student’s paired two-tailed *t*-tests, follows the assignment: * *p* < 0.05, ** *p* < 0.01, *** *p* < 0.001, and **** *p* < 0.0001.

As toxicity is a critical factor in selecting a suitable and effective antibacterial agent in various fields, especially in the textile industry, the cytotoxicity of GPEIs-functionalized oxCNDs was, initially, assessed against the Cos-7 normal African green monkey kidney cell line. Thus, cells were incubated with oxCNTs@GPEI5K and oxCNTs@GPEI5K at various concentrations for 24 h and then cell viability was evaluated, using the MTT assay. The results are presented in Figure 1. As can be observed, both hybrid nanomaterials do not exhibit any significant toxicity (cell survival ~70% at the higher tested concentrations 200 μg/mL after 24 h treatment) and therefore, their antibacterial properties were worth evaluating.

The antibacterial properties of oxCNTs@GPEIs as well as the parent oxCNTs were evaluated against Gram (-) *E. coli* and Gram (+) *S. aureus* bacteria. Specifically, their IC*_50_* and MIC values were established by the broth macro-dilution methods [54], while their MBC values were determined by the colony counting method [55]. The obtained results are presented in Table 1. Both oxCNTs@GPEIs show improved antibacterial activity compared to the parent oxCNTs, with oxCNTs@GPEI5K being more active than oxCNTs@GPEI25K. In detail, oxCNTs do not exhibit any antibacterial activity against both tested bacteria strains, as their MIC and MBC values are higher than 600 μg/mL, in agreement with the literature [19,20]. Furthermore, IC*_50_*, MIC and MBC values of both oxCNTs@GPEIs range from 50 to 100 μg/mL, 100 to 200 μg/mL, and 200 to 300 μg/mL respectively, for *E. coli* bacteria, while for *S. aureus* bacteria they range from 50 to 100 μg/mL, 80 to 150 μg/mL, and 150 to 250 μg/mL, respectively (Table 1), indicating a significant inhibitory activity against both strains. Based on these results, both nanohybrids could be classified as bactericidal materials as their MBC/MIC ratios are between 1 and 2 [55]. However, as the nanohybrid oxCNDs@GPEI5K exhibits better antibacterial activity simultaneously with lower toxicity (cell viability ~80% at MBC values i.e., 150–200 μg/mL), it can be considered a more promising antibacterial agent than oxCNDs@GPEI25K (cell viability > 60% at MBC values i.e., 250–300 μg/mL). Analogous results were obtained when the GPEIs derivatives were used for the functionalization of oxCNDs [51], in agreement with the literature reports noting that graphene functionalized with polyxamethylene guanidine hydrochloride exhibited enhanced antibacterial properties [71,72]. Hence, it is obvious that GPEIs offer enhanced antibacterial activity to oxCNTs as it is known that guanidinium groups can strongly interact with anionic phosphate and carboxylate groups of the bacterial walls through a combination of electrostatic interaction and bidentate hydrogen bonds, resulting in cell wall disruption or effective cell internalization leading to cell death, as they can affect bacterial metabolism due to their interaction with bacterial DNA, ribosomes and proteins [73,74].

Another important factor contributing to the improved antibacterial efficiency of oxCNTs@GPEIs is their superior aqueous dispersibility and stability compared to oxCNTs, which can enhance their interaction with bacteria, leading to increased cell damage. Research indicates that carbon-based materials such as carbon nanotubes [15], carbon nanodisks [51] or graphene oxide [72] that are well-dispersed, can significantly interact with bacteria through direct contact, leading to considerable damage to their membrane.

**Figure 2:**
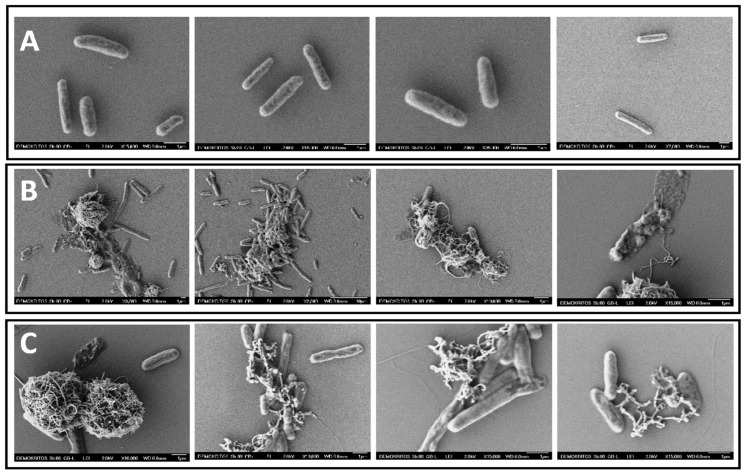
SEM images of *E. coli* bacteria: untreated cells (**A**) and cells after a 12-h incubation time at 37 °C with oxCNTs@GPEI5K (**B**) and oxCNTs@GPEI25K (**C**) at IC*_50_*.

The morphology of *E. Coli* bacteria following treatment with oxCNTs@GPEIs for a 12-h incubation time at 37 °C was studied using scanning electron microscopy (SEM). Figure 2 displays the SEM images of both untreated bacteria (control) and bacteria treated with oxCNTs@GPEIs at IC*_50_*. Untreated bacteria (Figure 2A) appear to be healthy having a typical rod shape with a smooth surface. In contrast, bacteria treated with both nanohybrids appear to have lost their cellular integrity seemingly resulting in bacterial death, as their surface is rougher and some of their cell walls and membranes have ruptured, causing intracellular components to leak out (Figure 2B,C). Also, as observed, nanohybrids cover and wrap the bacteria, resulting in the formation of CNTs-bacteria aggregates, which possibly inhibitthe uptake of nutrient from the surrounding environment and prevent cell proliferation, presumably leading to bacterial death. Remarkably, some bacteria seem to be elongated, which is a typical response of bacteria to stress.

Multi-walled CNTs are known not to exhibit any significant antibacterial activity due to their high tendency to aggregate, which inhibits their interactions with bacteria [8,43,47]. On the contrary, modified MWCNTs with various positive charged groups e.g., amines, arginines, lysines, etc. [75,76] or with various positive charged functional polymers [15,77,78,79] have been known to show improved antibacterial activities, due to their enhanced aqueous dispersibility and stability, which enable the direct contact between CNTs and bacteria. This leads to bacterial death either by blocking the transmembrane electron transfer and the nutrient uptake or by causing penetration that results in the rupture or distortion of cell envelope, thereby affecting the bacterial metabolic mechanisms. On the other hand, the antibacterial mechanism of action of polycationic molecules, particularly the guanidinylated polymers, is related to their capacity to strongly interact with the anionic moieties of the bacterial envelop through electrostatic interactions and hydrogen bonds, facilitating their translocation through bacterial membranes and their interaction with the divalent cations of membranes [80,81,82]. This results in disruption or rupture of membranes and cell walls, leakage of bacterial cytoplasmic components, and eventually bacterial death. In this work, GPEI derivatives provide MWCNTs with high water compatibility, inhibiting their agglomeration. Thus, the antibacterial activity of oxCNTs@GPEIs can be attributed to the synergetic effect of both the debundled MWCNTs and the GPEI derivatives that both interact strongly with bacteria, penetrating though cell envelops, thus leading to cell lysis and death.

### 3.2. Finishing of Textiles with oxCNTs@GPEI5K

In light of antibacterial and cytotoxic results, oxCNTs@GPEI5K was selected as finishing material for wool fabrics. Hybrid wool textiles (HT) with various oxCNTs@GPEI5K content raging between 1.9 to 8% wt. were prepared (Table 2). For comparison reasons GPEI5K and oxCNTs were applied as finishing materials on wool fabrics under the same procedure.

**Figure 3:**
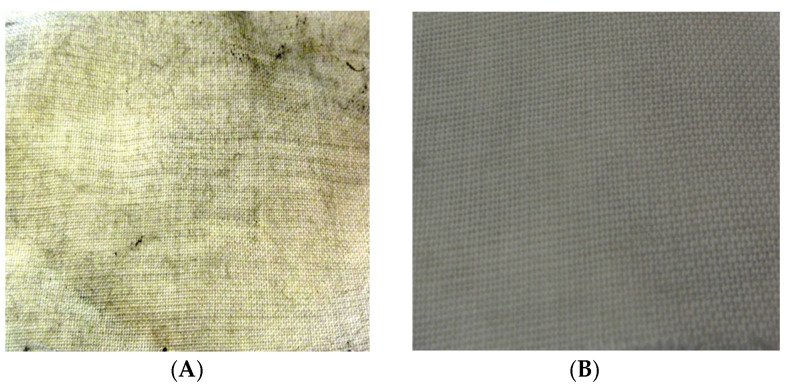
Optical images of: (**A**) HT_oxCNTs_ containing 8% wt. oxCNTs, and (**Β**) HT_oxCNTs@GPEI5K_ containing 8% wt. oxCNTs@GPEI5K.

As seen in Table 2, GPEI5Kcontent in HT_GPEI5K_ was very low indicating poor adhesion of the polymer to the wool fibers, while HT_oxCNTs_ samples were unevenly colored indicating uneven oxCNTs finish coverage (Figure 3), hence GPEI5K or oxCNTs alone cannot be considered suitable for textiles modification. On the other hand, HT_oxCNTs@GPEI5K_ samples with various oxCNTs@GPEI5K loadings are homogeneous, even at the higher tested oxCNTs@GPEI5K content, as shown in Figure 3. This indicates that oxCNTs@GPEI5K was uniformly adsorbed onto the wool fibers.

### 3.3. Chromatometric Analysis

Chromatometric analysis were used to investigate the effect of the treatment with various oxCNTs@GPEI5K loadings on the color appearance of fabrics. The color properties of all HT_oxCNTs@GPEI5K_ samples were assessed based on the CIE L*a*b* color space. Specifically, the L* indicates the lightness–darkness values from 100 to 0, corresponding from white to black, a* values run from negative (green) to positive (red) and b* values run from negative (blue) to positive (yellow) and the total color difference is given as ΔE* relative to the raw wool. In Table 3, the colorimetric measurement data for HT_oxCNTs@GPEI5K_ with increasing oxCNTs@GPEI5K content are presented. It is obvious that that color of functionalized wool fabrics significantly changed as a function of oxCNTs@GPEI5K content. Specifically, the color yield (*K*/*S*) values increase proportionally to the oxCNTs@GPEI5K content, with saturation beginning at dry wool containing 6.5% wt. oxCNTs@GPEI5K. This is also indicated by the Color Difference (ΔE*) and L* color coordinate values, where no significant change can be observed for oxCNTs@GPEI5K contents higher than 6.5% wt. Due to the oxCNTs@GPEI5K wool samples gray hue, an L* decrease of about 60% was observed at dry wool sample containing 8% wt. oxCNTs@GPEI5K.

### 3.4. Determination of Fastness Properties

For the determination of color fastness properties of functionalized HT samples, the dry wool sample containing 8% wt. oxCNTs@GPEI5K was selected. Wash fastness ratings for the HT_oxCNTs@GPEI5K-8%wt._ after washing procedures with standard soap, dry cleaning and liquid CO_2_ and their colorimetric characteristics are shown in Table 4.

A reduction of relative color strength (*RCS*) and an increase of color difference value (ΔE*) is observed after five standard soap (ISO 105-C10) washing cycles, due to oxCNTs@GPEI5K partially removal. This corresponds to a wash fastness rating of 4 (very good). On the other hand, samples subjected to ISO 105-D01 dry cleaning (5 cycles) or liquid CO_2_ treatment (equivalent of 50 cycles) exhibit excellent behavior with ΔE* values indicating very slight loss of oxCNTs@GPEI5K that corresponds to wash fastness rating of 5.

### 3.5. Evaluation of Antibacterial Properties

**Figure 4:**
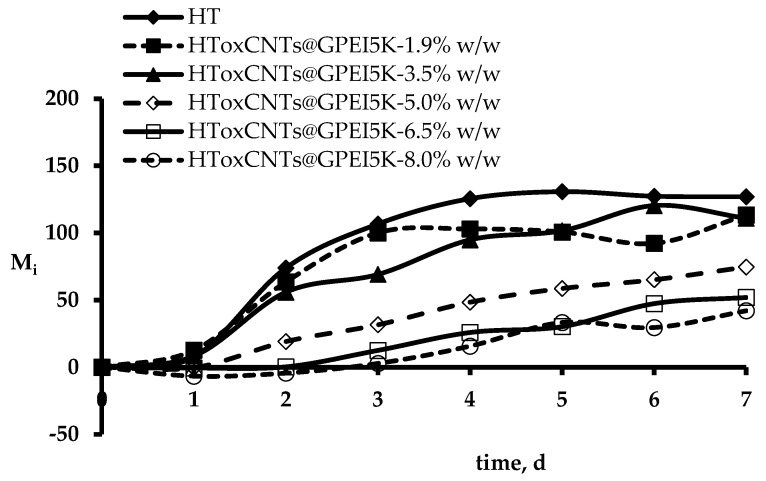
% change (*M*i) in Chl *α* fluorescence value of *Synechococcus* sp. PCC7942 incubated on HT_oxCNTs@GPEI5K_.

As mentioned, in order to assess the antibacterial properties of the as prepared HTs, a novel and easily applied method based on the quantifiable relationship of Chl α fluorescence (*F*_0_) and Cyanobacteria growth was used [65]. The method employs *Synechococcus* sp. PCC7942 to monitor the differences between bacterial growth on raw wool and on HT wool samples, by comparing the change in Chl α fluorescence every 24 h for seven days in an incubator under white fluorescent light (100 μE·m^−2^·s^−1^) at 31 °C. Figure 4 shows cyanobacterial Chl *α* fluorescence changes (*M*_i_) on HT_oxCNTs@GPEI5K_ adapted cells.

The *F*_0_ changes that correspond to inhibition of bacterial growth allow quantification through the calculation of the Bacterial Protection Index *Π*_7_ for each HT_oxCNTs@GPEI5K_ concentration, as described in Section 2.8 and the *Π*_7_ values after seven days of incubation are presented in Table 5.

Figure 4 shows that increasing oxCNTs@GPEI5K content results in increased stress for the test organisms compared to raw wool. Specifically, and looking also at the Bacterial Protection Index *Π*_7_, the antibacterial protection offered by oxCNTs@GPEI5K is significant.

**Figure 5:**
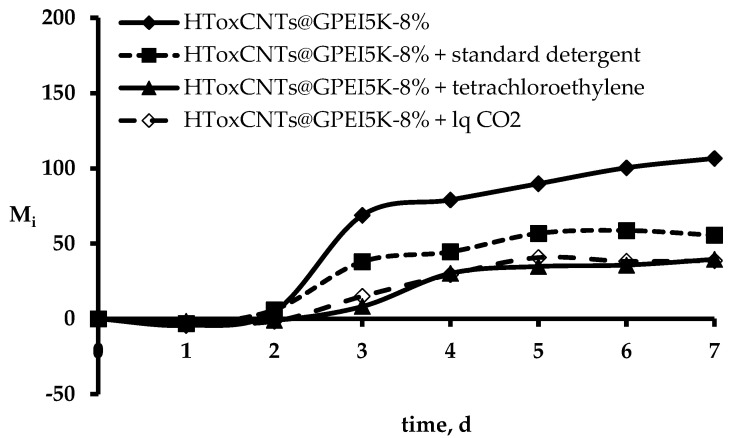
% change (*M*i) in Chl *α* fluorescence value of cyanobacterium *Synechococcus* sp. PCC7942 adapted on HT in the presence of oxCNTs@GPEI5K after washing.

In order to determine the durability of bacterial protection to washing treatments, the antibacterial performance of HT_oxCNTs@GPEI5K-8%wt._ sample was evaluated after washing. Figure 5 represents the *F*_0_ difference of cyanobacterial Chl *α* on wool samples subjected to different washing treatments with corresponding *Π*_7_ values presented in Table 6.

A decrease in *Π*_7_ is observed for the sample washed with standard soap (ISO 105-C10—5 cycles), while no significant changes are observed after dry-cleaning (ISO 105-D01—5 cycles) or liquid CO_2_ treatment (50 cycles). These results indicate that oxCNTs@GPEI5K nanohybrid attaches strongly onto the wool surface, forming a hybrid textile material that retains its antibacterial properties even after washing.

### 3.6. Bacteria SEM Analysis

**Figure 6:**
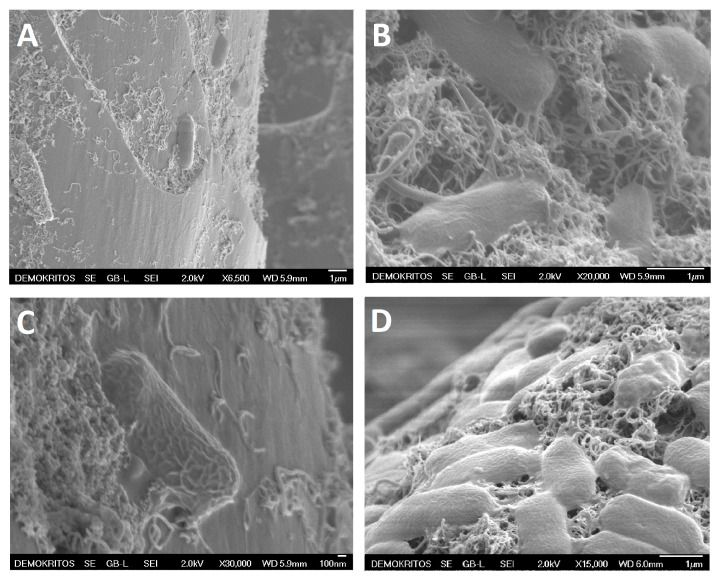
SEM images of cyanobacteria *Synechococcus* sp. PCC 7942 grown on HT_oxCNTs@GPEI5K-8%wt._

Figure 6A,B, shows SEM images of cyanobacteria grown on HT_oxCNTs@GPEI5K-8%wt._ fibers. oxCNTs@GPEI5K are highly dispersed on the fibers’ surface, to such an extent that individual CNTs can be distinguished. In Figure 6C,D cyanobacteria can be seen on the surface of the fibers. Some have already been destroyed as their shape has charged and others are in contact with CNTs, which are attached to the outer surface of the cells or have penetrated their cell wall.

### 3.7. Ultraviolet Radiation Blocking

**Figure 7:**
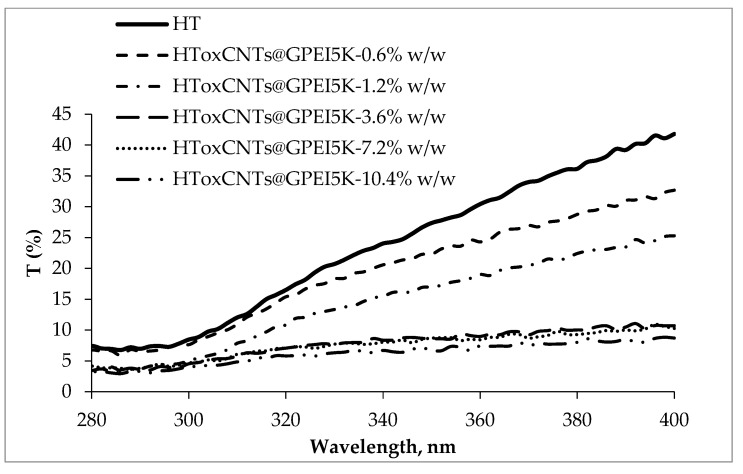
Transmittance of UV radiation through the HT_oxCNTs@GPEI5K_ compared to the untreated wool sample.

To determine the HTs UV radiation protection performance, the UPF values of treated and untreated wool fabrics were calculated according to the AATCC Test Method 183. The ultraviolet radiation transmission (UV-R) measurements and spectral data through HT_oxCNTs@GPEI5K_ are presented in Figure 7. It appears that there is a gradual decrease in transmission with increasing oxCNTs@GPEI5K content up to 5% wt., where a significantly high reduction in transmission is achieved. Further increase of oxCNTs@GPEI5K content does not significantly change the % Transmittance.

For further confirmation, ultraviolet protection factor (UPF), UV-A transmittance (315–400 nm), UV-B transmittance (280–315 nm) and percent UV-A and UV-B region blocking were measured according to the AATCC test method and are presented in Table 7.

While UV-A blocking increased from 70.9 to 92.9 (30.9%), the already high UV-B blocking value (90.8) for the untreated sample is increased by 5.8% reaching 96.1. On the other hand, the UPF value increased by 168.8% reaching 20.3 for the HT_oxCNTs@GPEI5K-8%wt._ sample. This value is characterized as “good”, according to the AS/NZS 4399 standard [83]. Tightly woven wool fabrics offer considerable UV protection due to their significant UV absorption properties with tricot knit fabric reaching a UPF value of about 45 [84]. However, the fabric used in this study was woven loosely, hence its relatively low UPF value (7.6). The significant UPF increase due to the addition of oxCNTs@GPEI5K indicates the important role played by CNTs in UV radiation absorption [85,86].

## 4. Conclusions

With a view to the preparation of multifunctional wool textiles, oxidized multi-walled carbon nanotubes (oxCNTs) functionalized with two guanidinylated hyperbranched polyethyleneimine (GPEI) derivatives (oxCNTs@GPEI5K and oxCNTs@GPEI25K) having enhanced aqueous stability and significant antibacterial properties, were used. Both nanohybrid materials exhibited potent antibacterial activity against Gram (-) and Gram (+) bacteria, with oxCNTs@GPEI5K demonstrating superior efficacy and lower cytotoxicity. The antibacterial mechanism was attributed to a combination of strong electrostatic interactions, cell wall penetration, and improved dispersion, which facilitated direct bacterial contact. Based on these findings, oxCNTs@GPEI5K was applied as a finishing material for wool fabrics, achieving uniform distribution and good adhesion. The resulting modified wool textiles exhibited improved ultraviolet protection and sustained antibacterial properties even after multiple washing cycles, making them excellent candidates for their use in antibacterial and UV-shielding textile applications. These results also highlight the versatility of guanidinylated oxCNTs nanohybrid as promising agent for biomedical and textile applications, providing a strong foundation for further research and their potential commercial utilization.

## Figures and Tables

**Table 1:** MIC and MBC values of oxCNTs and oxCNTs@GPEIs against *E. coli* and *S. aureus* bacteria.

Samples	Gram (-) *E. coli* Bacteria			Gram (+) *S. aureus* Bacteria		
	IC*_50_* (μg/mL)	MIC (μg/mL)	MBC (μg/mL)	IC*_50_* (μg/mL)	MIC (μg/mL)	MBC (μg/mL)
oxCNTs	400	>600	N/A	>600	600	N/A
oxCNTs@GPEI5K	50	100	200	10	80	150
oxCNTs@GPEI25K	100	200	300	20	150	250

**Table 2:** GPEI5K, oxCNTs and oxCNTs@GPEI5K content on wool substrate.

Sample	% wt. Dry Wool				
HT_GPEI5K_	0.05	0.08	0.11	0.13	
HT_oxCNTs_	1.9	3.5	5	6.5	8
HT_oxCNTs@GPEI5K_	1.9	3.5	5	6.5	8

**Table 3:** Colorimetric characterization for HT/oxCNTs@GPEI5K samples.

**oxCNTs@GPEI5K** **% wt. Dry Wool**	***K*** **/** ***S***	***RCS* (%)**	***L*** *	***α*** *	***b*** *	**Δ** ***E*** *
0 (raw wool)	0.323	100.0	85.56	−0.35	12.06	-
1.9	0.569	176.1	70.62	−0.51	6.62	15.91
3.5	0.979	303.2	64.81	−0.36	5.40	21.79
5	2.923	904.9	44.97	0.10	2.34	41.75
6.5	5.290	1637.8	35.92	0.21	2.09	50.64
8	5.722	1771.5	34.51	0.12	1.75	52.09

**Table 4:** Colorimetric characterization and wash fastness ratings of the dry wool sample containing 8% wt. oxCNTs@GPEI5K (HT_oxCNTs@GPEI5K-8%_).

	*K*/*S*	*RCS* (%)	L*	α*	b*	ΔE*	Wash Fastness Rate
HT_oxCNTs@GPEI5K-8%_	5.722	100.0	-	-	-	-	-
HT_oxCNTs@GPEI5K-8%_ after washing with ISO 105-C10 (B2)	5.010	87.6	1.98	−0.20	−0.11	1.99	4
HT_oxCNTs@GPEI5K-8%_ after washing with ISO 105-D01	5.692	99.5	0.19	−0.01	0.08	0.21	5
HT_oxCNTs@GPEI5K-8%_ after washing with liquid CO_2_	5.711	99.8	0.12	−0.01	0.06	0.13	5

**Table 5:** HTs Bacterial Protection Index *Π*_7._

Content % wt. dry wool	0	1.9	3.5	5	6.5	8
*Π*_7_	7.1	10.7	12.6	41.2	59.1	66.9

**Table 6:** Bacterial Protection Index *Π*_7_ of HT_oxCNTs@GPEI5K-8%wt._ sample after washing.

	Before washing	Standard soap	Dry clean	Liquid CO_2_
*Π_7_*	66.9	47.8	62.9	63.7

**Table 7:** UV blocking data (according to AATCC Test Method 183) for HT_oxCNTs@GPEI5K_ samples.

% wt. oxCNTs@GPEI	Blocking UV-A (%)	Blocking UV-B (%)	UPF	UPF Rate ^1^
0 (raw wool)	70.9	90.8	7.6	insufficient
1.9	76.3	91.5	8.4	insufficient
3.5	81.9	94.5	12.0	insufficient
5	91.0	95.4	16.9	good
6.5	91.4	95.2	16.9	good
8	92.9	96.1	20.3	good

^1^ UPF rate according to AS/NZS 4399 standard.

## Data Availability

The data presented in this study are available on request from the corresponding author.

## Supplementary Materials

The following supporting information can be downloaded at: https://www.mdpi.com/article/10.3390/ma18091993/s1, Figure S1: ^1^H NMR spectra (500 MHz, D_2_O) of GPEI5K (A) and GPEI25K (B); Figure S2. ^13^C NMR spectra (125.1 MHz, D_2_O) of GPEI5K (A) and GPEI25K (B); Table S1. Elemental analysis results of oxCNTs, GPEI and GPEI-functionalized oxCNTs; Details for the calculation of polymer loading.
