# Supplementary material for: Fabrication of Antibacterial and Ultraviolet Protective Wool Fabric Using Multi-Walled Carbon Nanotubes Functionalized with Guanidinylated Hyperbranched Polyethyleneimine Derivative
Source: Materials (Basel). 2025 Apr 28;18(9):1993. doi: 10.3390/ma18091993 (PMC12072795; doi:10.3390/ma18091993)
Supplement: Supplementary file 1 [file materials-18-01993-s001.zip › materials-3564335-supplementary.pdf]

Supporting information

Fabrication of antibacterial and ultraviolet protective wool fabric using multi-walled carbon nanotubes functionalized with guanidinylated hyperbranched polyethyleneimine derivative

Nikolaos S. Heliopoulos [1,2], Kyriaki-Marina Lyra [1], Aggeliki Papavasiliou [1], Fotios K. Katsaros [1], Kostas Stamatakis [3], Sergios K. Papageorgiou [1,*] and Zili Sideratou [1,*]

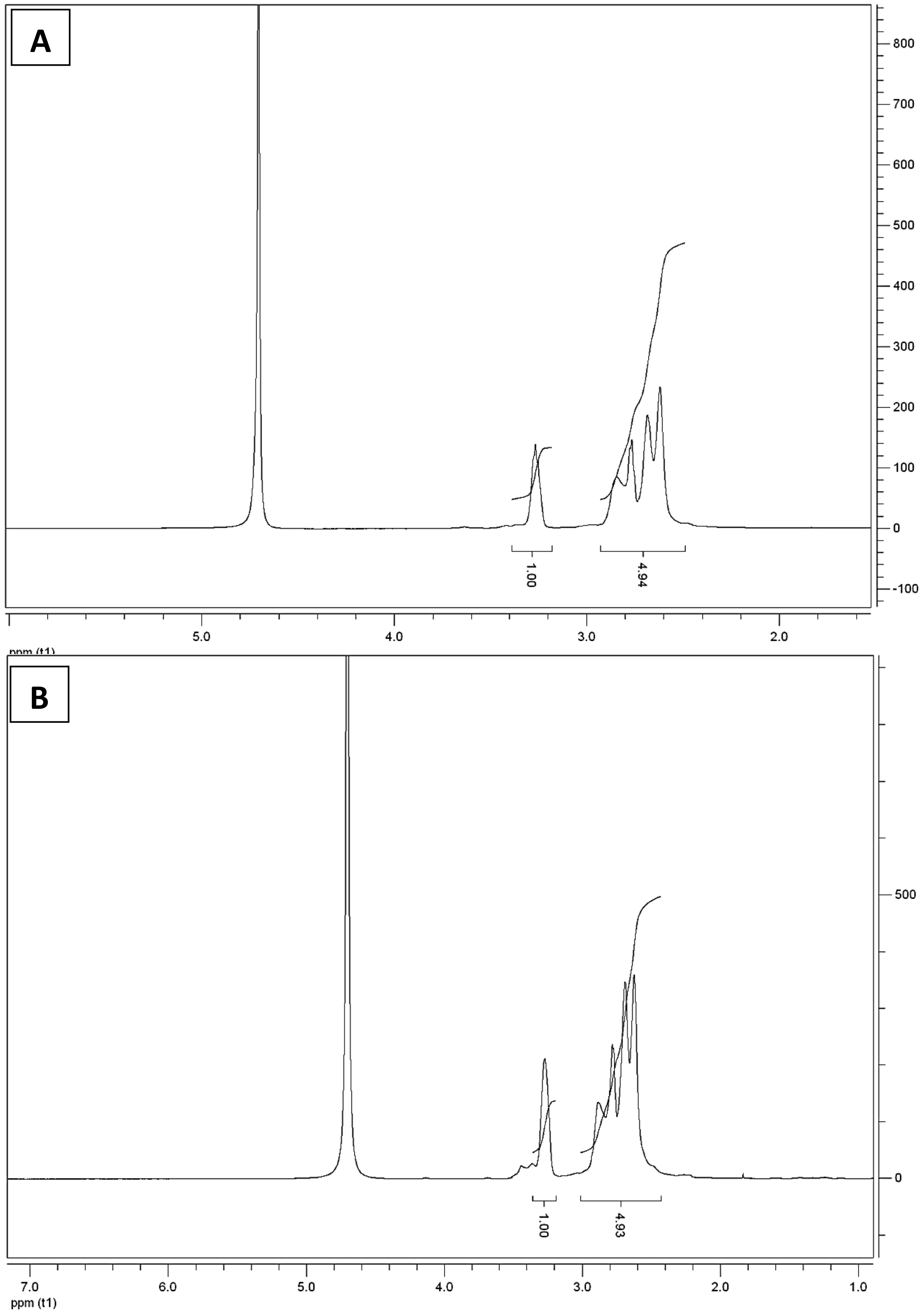

Figure S1. ^{1}H NMR spectra (500 MHz, D_2O) of GPEI5K (A) and GPEI25K (B).

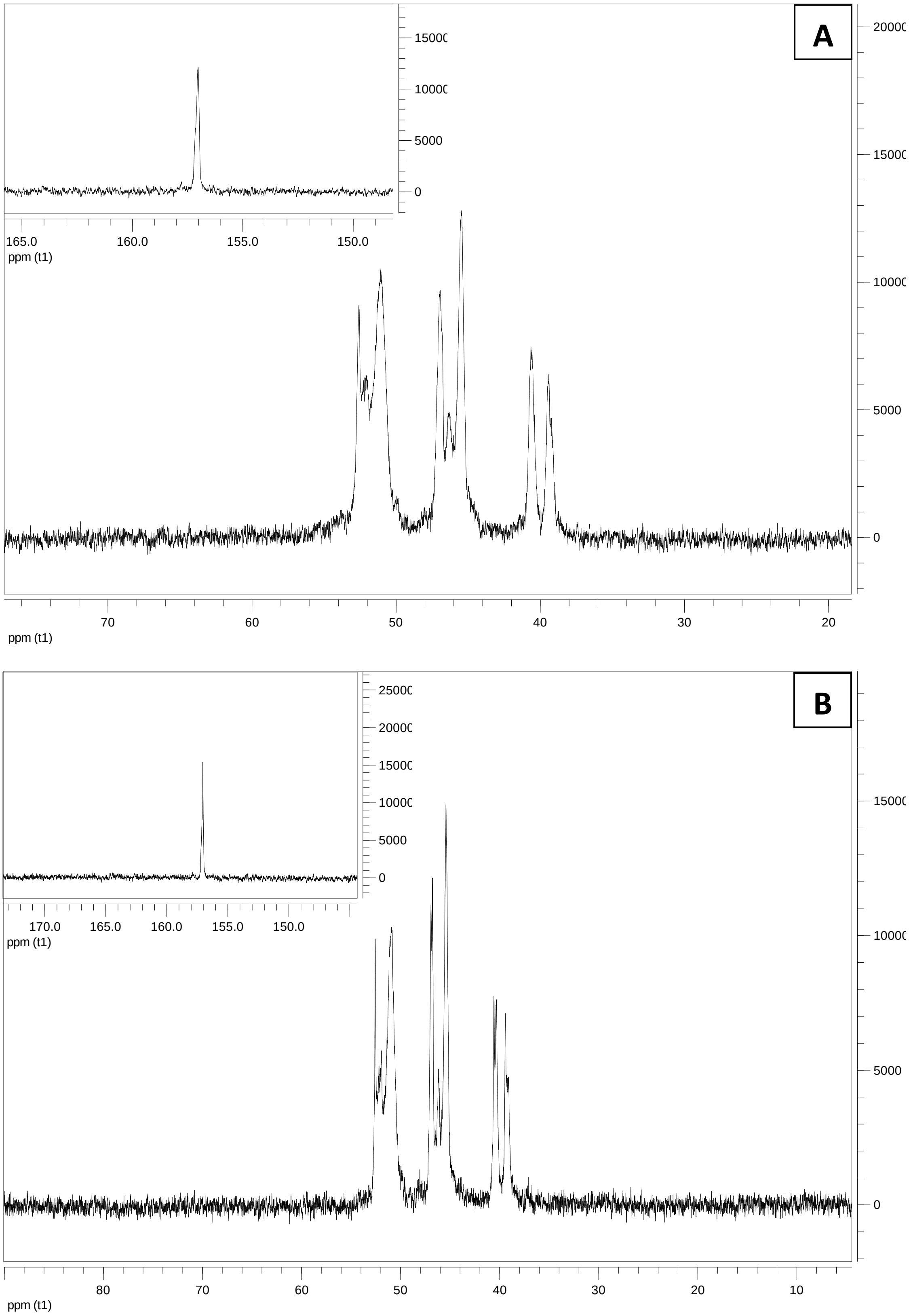

Figure S2. ^{13}C NMR spectra (125.1 MHz, D_2O) of GPEI5K (A) and GPEI25K (B).

Calculation of polymer loading

To determine the GPEIs content in ox-CNTs@GPEI5K and oxCNTs@GPEI25K hybrids, elemental analysis (EA) was applied using a Perkin Elmer 240 CHN elemental analyzer. As the nitrogen signal in the functionalized oxCNTs mainly originates from GPEI, the quantity of GPEIs attached to the oxCNTs can be calculated by comparing the nitrogen signal of the final materials to that of the starting oxCNTs using the following formula:

$$GPEI\ (\%\ w/w) = (N_s - N_{oxCNTs})/(N_{GPEI} - N_{oxCNTs})*100$$

where N_s, N_{GPEI} and N_{oxCNTs}, are the nitrogen elemental mass fraction in GPEI-functionalized oxCNTs, GPEI and oxCNTs, respectively. The results are summarized in Table S1.

Table S1. Elemental analysis results of oxCNTs, GPEI and GPEI-functionalized oxCNTs.

	**Sample elemental composition/wt%**			
Sample	C	H	N	GPEI (% w/w)
oxCNTs	91.52	1.86	0.23	
GPEI5K	35.63	10.26	32.32	
oxCNTs@GPEI5K	69.54	2.08	7.51	22.7%
GPEI25K	38.19	8.52	33.56	
oxCNTs@GPEI25K	59.29	1.51	9.40	27.5 %
